# Detection of cardiac neuroendocrine tumour metastases by somatostatin receptor PET/CT: a systematic review and meta-analysis

**DOI:** 10.3389/fmed.2024.1491181

**Published:** 2024-10-15

**Authors:** Daniela Campanale, Alessio Imperiale, Domenico Albano, Alessio Rizzo, Arnoldo Piccardo, Giorgio Treglia

**Affiliations:** ^1^Division of Cardiology, Istituto Cardiocentro Ticino, Ente Ospedaliero Cantonale, Bellinzona, Switzerland; ^2^Division of Nuclear Medicine and Molecular Imaging, Institut de Cancérologie de Strasbourg Europe (ICANS), Strasbourg University Hospitals, Strasbourg, France; ^3^Molecular Imaging, DRHIM, Institut Pluridisciplinaire Hubert Curien (IPHC), UMR7178, CNRS, University of Strasbourg, Strasbourg, France; ^4^Division of Nuclear Medicine, Università degli Studi di Brescia and ASST Spedali Civili di Brescia, Brescia, Italy; ^5^Division of Nuclear Medicine, Candiolo Cancer Institute, FPO-IRCCS, Turin, Italy; ^6^Division of Nuclear Medicine, Galliera Hospital, Genoa, Italy; ^7^Department of Nuclear Medicine and Molecular Imaging, Lausanne University Hospital and University of Lausanne, Lausanne, Switzerland; ^8^Division of Nuclear Medicine, Imaging Institute of Southern Switzerland, Ente Ospedaliero Cantonale, Bellinzona, Switzerland; ^9^Faculty of Biomedical Sciences, Università della Svizzera italiana, Lugano, Switzerland

**Keywords:** meta-analysis PET/CT, positron emission tomography, hybrid imaging, cardiac metastases, neuroendocrine tumours, neuroendocrine neoplasm, somatostatin, nuclear medicine

## Abstract

**Background:**

Cardiac neuroendocrine tumour metastases (CNTM) are rare, but advancements in molecular imaging including somatostatin receptor PET/CT (SSTR-PET/CT) could lead to a more frequent identification. The aim of this article is to perform a systematic review and meta-analysis on the detection of CNTM by SSTR-PET/CT.

**Methods:**

A comprehensive literature search of studies on CNTM detected by SSTR-PET/CT was carried out. Three different bibliographic databases were screened (Cochrane library, PubMed/MEDLINE, EMBASE) until 20 August 2024. Two review authors independently selected the eligible original articles and performed the quality assessment and the data extraction. Main findings of eligible studies were summarized and a proportion meta-analysis on the prevalence of patients with CNTM among those with neuroendocrine neoplasm (NEN) performing SSTR-PET/CT was carried out using a random-effects model.

**Results:**

Ten articles reporting data on 163 patients with CNTM were included in the systematic review. SSTR was able to detect CNTM earlier compared to other radiological imaging techniques. Most patients with CNTM had other metastatic sites and CNTM were often asymptomatic. The meta-analysis of seven articles demonstrated a pooled prevalence of 1.5% (95% confidence interval: 1.0–1.9%) of patients with CNTM (*n* = 119) among those performing SSTR-PET/CT for NEN (*n* = 9,300). Moderate statistical heterogeneity was found (*I*^2^ test: 62%).

**Conclusion:**

Evidence-based data demonstrate that SSTR-PET/CT enables early and better detection of CNTM compared to other radiological imaging methods. CNTM are encountered with a pooled prevalence of 1.5% of NEN patients performing SSTR-PET/CT. Prospective and multicentric studies are warranted to better clarify the impact of CNTM detection by SSTR-PET/CT on overall survival and clinical decision-making in NEN patients.

## Introduction

Neuroendocrine neoplasms (NEN) include heterogeneous tumours arising from neuroendocrine cells. Most NEN are well-differentiated neuroendocrine tumours with indolent disease biology, whereas poorly differentiated NEN with rapid disease progression are less frequent (1). The incidence and prevalence of NENs continues to rise globally and gastroenteropancreatic NEN represent the most common subtype. Metastatic disease from NEN is frequent and the most frequent metastatic sites are lymph nodes liver, and bone (1).

Cardiac neuroendocrine tumours metastases (CNTM) are rare with an estimated incidence lower than 1% (1, 2). However, the incidence of CNTM is likely underestimated as they often remain undetected until autopsy (1). CTNM are also difficult to diagnose due to their anatomic location and their small size especially in their early stages. Notably, CNTM can lead to a poor clinical outcome and their prompt diagnosis is relevant to start the most effective treatment and for their management (3).

Advancements in molecular imaging including somatostatin receptor PET/CT (SSTR-PET/CT) have led to more frequent identification of NEN lesions compared to conventional imaging including CT and magnetic resonance imaging (MRI). Evidence-based data have demonstrated that SSTR PET/CT (using somatostatin analogues radiolabelled with gallium-68 or copper-64) has high diagnostic performance in detecting NEN lesions due to their usual high SSTR expression (4–6).

**Figure 1:**
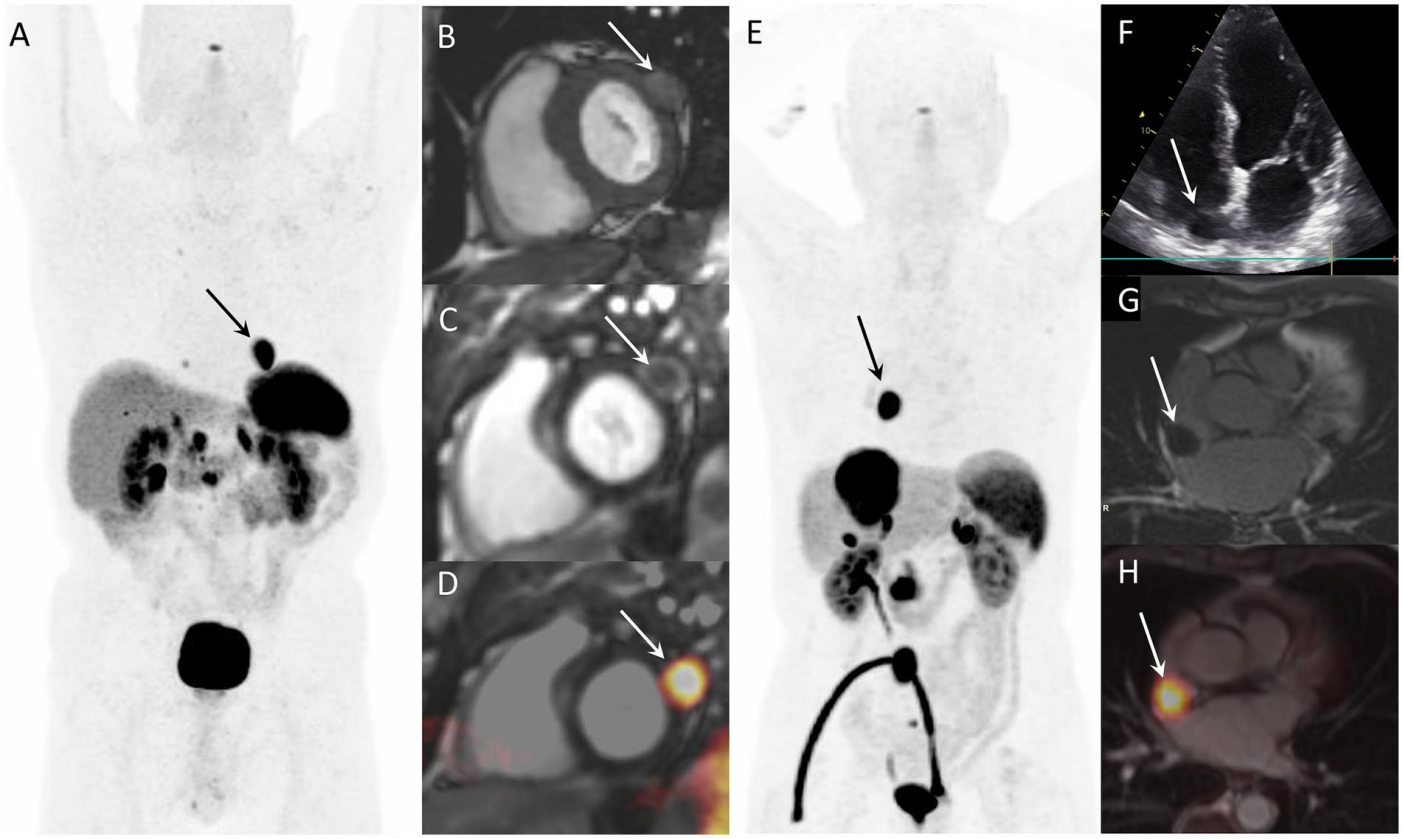
Examples of CNTM detected by SSTR-PET/CT in two NEN patients. Case 1: Myocardial metastasis of left ventricular latero-basal wall (arrows) detected by SSTR-PET/CT (A: anterior MIP) in a 74-year-old patient with G2 pancreatic NEN. The CNTM appeared at cardiac magnetic resonance (CMR) as a 22-mm nodule with intermediate intensity on T1-weighted images (B, short axis), with peripheral contrast-enhancement on phase sensitive inversion recovery sequence (C, short axis). Fused SSTR-PET/CMR image is also showed (D, short axis). Case 2: 76-year-old patient with inter-atrial myocardial lesion (arrows) of metastatic G1 small intestinal NEN detected by SSTR-PET/CT (E: anterior MIP). Echocardiography (4-chamber view) showed hypoechoic mass on the right side of interatrial septum (F). The CNTM showed a slight peripheral enhancement on delayed post-contrast inversion recovery CMR sequences (G, short axis). Fused SSTR-PET/CMR image is also showed (H, axial slice).

After the first case of CNTM detected by SSTR-PET/CT in 2010 (7), several articles and case series were published on this topic (3). Two examples of CNTM detected by SSTR-PET/CT are reported in Figure 1. We hypothesize that SSRT-PET/CT could lead to a more frequent identification of CNTM. Therefore, the aim of this evidence-based article is to perform a systematic review of the literature and a meta-analysis on the detection of CNTM by SSTR-PET/CT.

## Methods

### Protocol, review authors and review question

This article was written according to a predefined protocol for systematic reviews and meta-analysis of diagnostic tests (8) and following the updated version “Preferred Reporting Items for a Systematic Review and Meta-Analysis of Diagnostic Test Accuracy Studies” statement (9).

The working group was composed of one cardiologist (DC) and five nuclear medicine physicians or radiologists working in different European centers with experience in hybrid imaging in NEN and systematic reviews/meta-analyses (AI, DA, AR, AP, and GT).

The first step of the working group was to formulate a clear review question (“Which is the role of SSTR-PET/CT in detecting CNTM compared to other imaging methods?”) created using the following PICO (Patients/Intervention/Comparison/Outcome) framework:

- Patients: individuals with CNTM.
- Intervention: SSTR-PET/CT performed for NEN staging/restaging.
- Comparison: other imaging methods for detecting CNTM.
- Outcomes: in patients with NEN performing SSTR-PET/CT the outcomes evaluated were: prevalence of patients with CNTM, site of CNTM, tracer uptake of CNTM, presence of concurrent metastases, presence of cardiac symptoms, performance of SSTR-PET/CT in detecting CNTM compared to other radiological imaging methods, management of CNTM after SSTR-PET/CT.

### Search strategy

To reduce possible biases, two review authors (DC as junior researcher and GT as senior researcher) independently performed a comprehensive literature search using three different bibliographic databases (Cochrane library, PubMed/MEDLINE and EMBASE) to find published original articles on the role of SSTR-PET/CT in detecting CNTM. The following search string combining several free text key words was used: (A) “PET” OR “positron” AND (B) “somatostatin” OR “DOTA*” OR “neuroendocrine” OR “NET” OR “NEN” and (C) “cardiac” OR “myocardial” OR “heart.” Last date of the literature search was 20 August 2024. For a more comprehensive literature search no beginning date limit nor language restrictions were used and references of retrieved eligible articles were screened searching for additional studies.

### Study selection

Predefined inclusion criteria were: studies or subsets of studies investigating the role of SSTR-PET/CT in detecting CNTM. The predefined exclusion criteria were: (a) articles outside the field of interest of this review (b) review articles, letters, editorials, comments and conference proceedings in the field of interest of this review; (c) case reports (less than 4 patients with CNTM described) in the field of interest of this review.

Three review authors (DC, AR, and GT) applied the inclusion and exclusion criteria mentioned above and independently screened the titles and abstracts of the retrieved records using the predefined search string in the selected bibliographic databases. After the exclusion of non-eligible records, the same three researchers then independently screened the full-texts of the potential eligible articles. Eligible articles were included in the systematic review after a virtual consensus meeting of the whole working group to solve any possible disagreement. Articles included in the systematic review were included in the meta-analysis only when there was absence of patient data overlap and when both overall number of NEN patients performing SSTR-PET/CT and number of CNTM detected by SSTR-PET/CT were specified.

### Data extraction

Two review authors (DC and GT) independently extracted the following information from the selected eligible articles using predefined data collection forms: basic study characteristics (authors, year of publication, country of origin, study design and funding), patient characteristics (number and subtype of NEN patients performing SSTR-PET/CT, patients with CNTM detected by SSTR-PET/CT, male percentage and mean age of patients with CNTM, primary NEN site and grading in patients with CNTM), technical aspects (hybrid imaging modality, date of SSTR-PET/CT imaging, PET tracer and injected activity, time interval between tracer injection and image acquisition, image analysis, comparison with other imaging methods), outcome data in patients with NEN performing SSTR-PET/CT (prevalence of patients with CNTM, site of CNTM, tracer uptake of CNTM, presence of concurrent metastases, presence of cardiac symptoms, performance of SSTR-PET/CT in detecting CNTM compared to other radiological imaging methods, therapeutic management of CNTM after SSTR-PET/CT).

### Quality assessment

The overall quality of the studies included in this systematic review was performed using the QUADAS-2 tool (“Revised Quality Assessment of Diagnostic Accuracy Studies”) (10).

### Statistical analysis

We calculated through a meta-analysis the prevalence of patients with CNTM among those with NEN performing SSTR-PET/CT. This proportion meta-analysis was performed using a random-effects model which considers the variability between studies. Pooled data were presented with their respective 95% confidence interval (95%CI) values and displayed using a forest plot. Heterogeneity was estimated through the *I*-square (*I*^2^) or inconsistency index (11). The publication bias was assessed through the Egger’s test (12). Subgroup analyses were planned in case of significant statistical heterogeneity. OpenMeta[Analyst] was used as free open-source software for the meta-analyses.^1^

## Results

### Literature search

**Figure 2:**
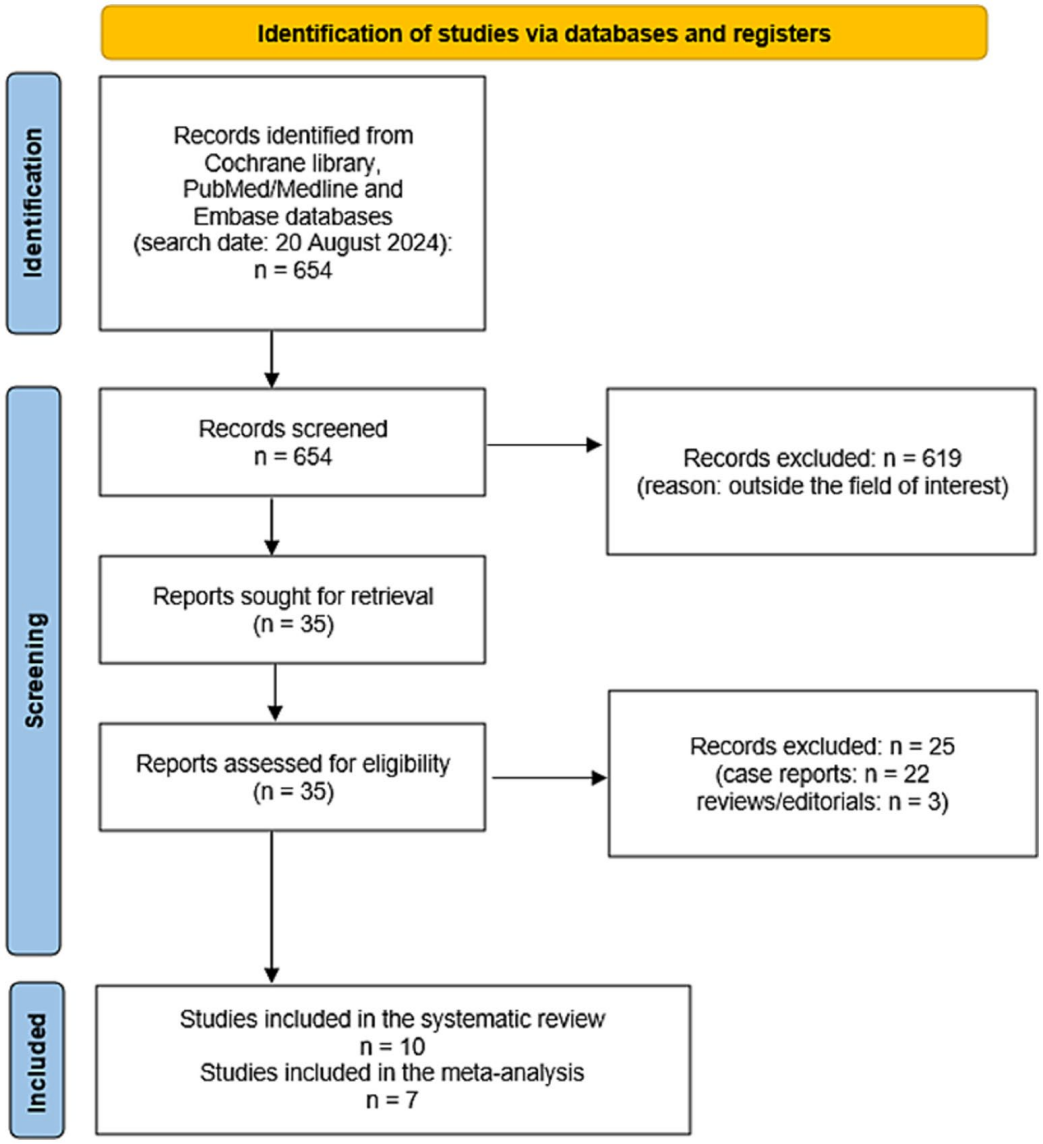
Flow chart of the search for eligible studies on the role SSTR-PET/CT for CNTM detection.

Figure 2 summarizes the results of the systematic literature search. A total of 654 records were identified using the selected databases. Titles and abstracts of 654 records were screened and 619 were excluded because they were outside the field of interest of this review. Then, the full text of 35 remaining records was screened and 25 articles were excluded (22 as case reports and 3 as reviews or editorials). Finally, 10 studies reporting data on 163 patients with CNTM were included in the systematic review (13–22) and no additional studies were found screening the reference list of the retrieved articles. Seven of these 10 studies were also included in the meta-analysis (13, 15, 18–22). Three studies were included in the systematic review but excluded from the meta-analysis because they did not provide sufficient information on the overall number of NEN patients performing SSTR-PET/CT (14, 16, 17).

### Qualitative analysis (systematic review)

**Figure 3:**
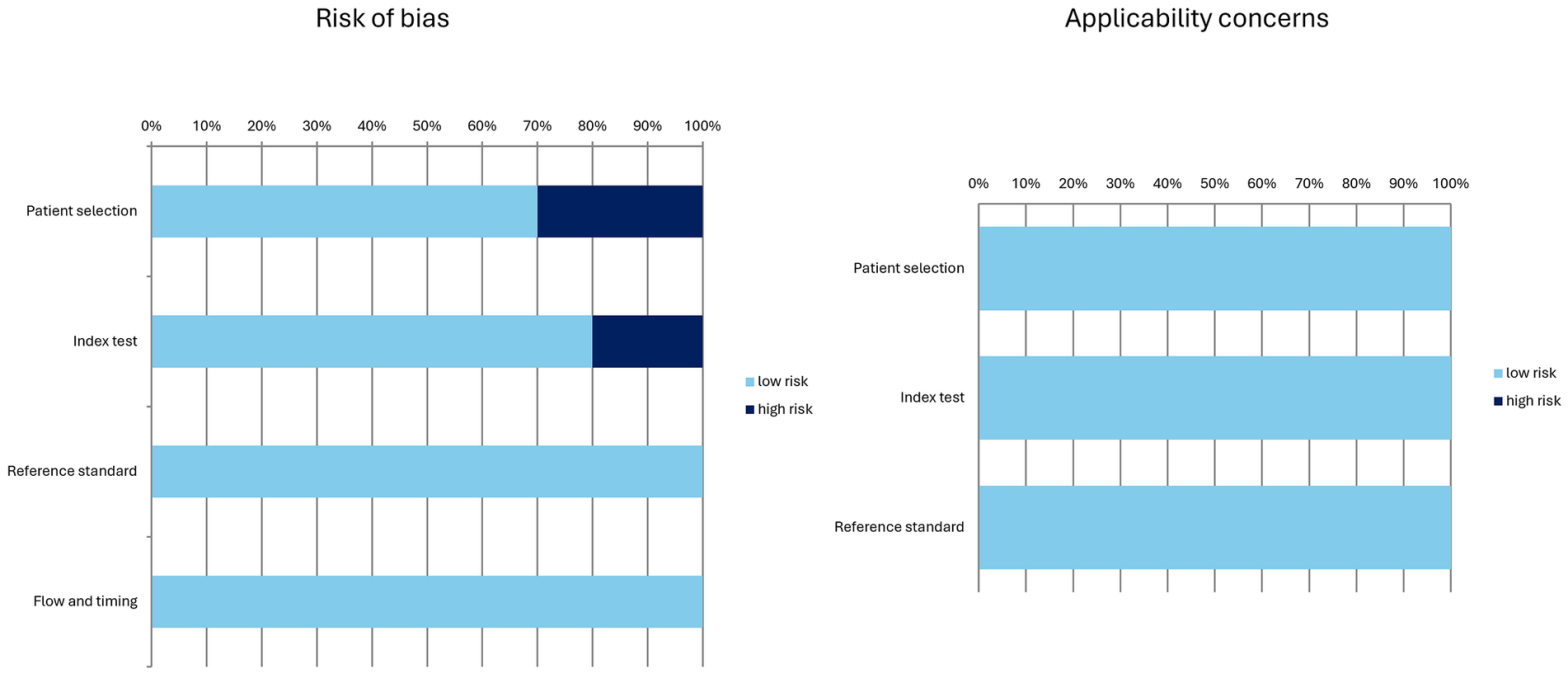
Overall quality assessment of the studies included in the systematic review according to the QUADAS-2 tool.

The characteristics of the included studies are presented in Tables 1–4. The overall quality assessment (including risk of bias and applicability concerns) of the 10 studies included in the systematic review is illustrated in Figure 3.

**Table 1:** Basic study characteristics of included studies.

Authors	Publication year	Country	Study design	Funding
Wedin et al. (13)	2024	Sweden	Retrospective multicentre	None reported
Arnfield et al. (14)	2023	Australia	Retrospective single centre	None reported
Wang et al. (15)	2023	USA	Retrospective single centre	American Heart Associate Award
El Ghannudi et al. (16)	2022	France	Retrospective single centre	None reported
Liu et al. (17)	2021	United Kingdom	Retrospective single centre	China Postdoctoral Science Foundation
Moyade et al. (18)	2019	United Kingdom	Retrospective single centre	None reported
Kunz et al. (19)	2018	Germany	Retrospective single centre	None reported
Bonsen et al. (20)	2016	Netherlands	Retrospective single centre	None reported
Calissendorff et al. (21)	2014	Sweden	Retrospective single centre	None reported
Carreras et al. (22)	2013	Germany	Retrospective single centre	None reported

**Table 2:** Patients’ characteristics of included studies.

Authors	Number of NEN patients performing SSTR-PET/CT	Subtype of NEN performing SSTR PET/CT	Patients with CNTM detected by SSTR-PET/CT	Male percentage among patients with CNTM	Mean age of patients with CNTM (years)	Primary NEN site in patients with CNTM	NEN grading in patients with CNTM
Wedin et al. (13)	1,171	Small intestinal and pancreatic NEN	15	NR	NR	Small bowel (93%), pancreas (7%)	NR
Arnfield et al. (14)	NR	Not specified	19	58%	63	Small bowel (79%), lung (21%)	Grade 1: 42% Grade 2: 26% Unknown: 32%
Wang et al. (15)	1,426	Gastroenteropancreatic NEN	25	56%	64	Small bowel (36%), colon (8%), pancreas (16%), mesentery (4%), unknown (36%)	Grade 1: 44% Grade 2: 20% Unknown: 36%
El Ghannudi et al. (16)	NR	Not specified	4	100%	74	Small bowel (100%)	Grade 1: 50% Grade 2: 50%
Liu et al. (17)	NR	Not specified	21	56%	64	Small bowel (84%), pancreas (4%), lung (4%), unknown (8%)	grade 1: 44% Grade 2: 36% Grade 3: 8% Unknown: 12%
Moyade et al. (18)	1,463	Not specified	19	74%	65	Small bowel (32%), colon (16%), pancreas (5%), paraganglia (5%), von Hippel–Lindau syndrome (5%), unknown (37%)	NR
Kunz et al. (19)	629	Not specified	15	60%	65	Small bowel (73%), colon (20%), unknown (7%)	Grade 1: 33% Grade 2: 20% Grade 3: 13% Unknown: 33%
Bonsen et al. (20)	273	Not specified	6	83%	54	Small bowel (50%), rectum (17%), ovary (17%), unknown (17%)	Grade 1: 66% Grade 2: 33%
Calissendorff et al. (21)	128	Ileal NEN	4	25%	62	Small bowel (100%)	Grade 1: 100%
Carreras et al. (22)	4,210	Not specified	35	NR	NR	NR	NR

CNTM, cardiac neuroendocrine tumour metastases; NEN, neuroendocrine neoplasm; NR, not reported; SSTR-PET/CT, somatostatin receptor positron emission tomography/computed tomography.

**Table 3:** Technical aspects of included studies.

Authors	Hybrid imaging modality	Date of SSTR-PET/CT scan	Type of tracer (mean injected activity)	Time interval between tracer injection and image acquisition	Image analysis	Other imaging modalities used for comparison
Wedin et al. (13)	PET/CT (contrast-enhanced CT)	01.2010–06.2022	[^68^Ga]Ga-DOTATOC (2 MBq/kg)	60 min	Visual	NR
Arnfield et al. (14)	PET/CT	01.2015–05.2020	[^68^Ga]Ga-DOTATATE (120–200 MBq)	45–75 min	Visual and semiquantitative (SUV_max_)	MRI, echocardiography
Wang et al. (15)	PET/CT	10.2017–03.2020	[^68^Ga]Ga-DOTATATE (185 MBq)	40 min	Visual and semiquantitative (SUV_max_)	CT, MRI, echocardiography
El Ghannudi et al. (16)	PET/CT	NR	[^68^Ga]Ga-DOTATOC (NR)	NR	Visual and semiquantitative (SUV_max_)	CT, MRI, echocardiography, [^18^F]FDOPA PET/CT
Liu et al. (17)	PET/CT	NR	[^68^Ga]Ga-DOTATATE (NR)	NR	Visual	CT, MRI, echocardiography, [^111^In]In-pentreotide SPECT/CT, [^18^F]FDG PET/CT
Moyade et al. (18)	PET/CT	2013–2018	NR (150–250 MBq)	60 min	Visual	NR
Kunz et al. (19)	PET/CT (contrast-enhanced CT)	03.2012–03.2017	[^68^Ga]Ga-DOTATATE (223 MBq)	60 min	Visual and semiquantitative (SUV_mean_ and SUV_max_)	CT, MRI
Bonsen et al. (20)	PET/CT	08.2011–06.2015	[^68^Ga]Ga-DOTATATE (NR)	NR	Visual	CT
Calissendorff et al. (21)	PET/CT (contrast-enhanced CT)	01.2010–04.2012	[^68^Ga]Ga-DOTATOC (135 MBq)	30 min	Visual and semiquantitative (SUV_mean_ and SUV_max_)	CT, echocardiography
Carreras et al. (22)	PET/CT	07.2004–12.2009	NR	NR	Visual	NR

[^18^F]FDG, fluorine-18 fluorodeoxyglucose; [^18^F]FDOPA, fluorine-18 dihydroxyphenylalanine; [^68^Ga]Ga-DOTATOC and [^68^Ga]Ga-DOTATATE, somatostatina analogues labelled with gallium-68; CT, computed tomography; MBq, megabequerel; min, minutes; MRI, magnetic resonance imaging; NR, not reported; PET, positron emission tomography; SPECT, single photon emission computed tomography; SSTR, somatostatin receptor; SUV_max_, maximal standardized uptake value; SUV_mean_, mean standardized uptake value.

**Table 4:** Outcome data of included studies.

Authors	Prevalence of patients with CNTM among NEN patients performing SSTR-PET/CT	Specific site of CNTM detected by SSTR-PET/CT	Mean SUV_max_ of CNTM (range)	Presence of concurrent metastases in patients with CNTM	Patients with CNTM without cardiac symptoms	Performance of other radiological imaging in detecting CNTM compared to SSTR-PET/CT	Management of CNTM
Wedin et al. (13)	15/1,171 (1.28%)	Left ventricle (60%), pericardium (20%), left atrium (13%), right ventricle (7%)	NR	Most cases	NR	NR	NR
Arnfield et al. (14)	19/NR (NC)	Left ventricle (59%), right ventricle (23%), septum (9%), other sites (9%)	7.5 (2–137.8)	Most cases	Most cases	Lower	PRRT + SSA (58%), SSA (21%), PRRT (5%), other (16%)
Wang et al. (15)	25/1,426 (1.75%)	Left ventricle (50%), septum (21%), right ventricle (19%), pericardium (12%)	9 (NR)	Most cases	Most cases	Lower	NR
El Ghannudi et al. (16)	4/NR (NC)	Left ventricle (75%), septum (25%)	25.6 (5.6–37.1)	Most cases	Most cases	Lower	SSA (75%), SSA + PRRT (25%)
Liu et al. (17)	21/NR (NC)	Left ventricle (52%), right ventricle (44%), pericardium (14%), septum (12%), left atrium (8%), right atrium (4%)	NR	All cases	Most cases	Lower	SSA (88%), PRRT (64%), other (40%)
Moyade et al. (18)	19/1,463 (1.3%)	Left ventricle (42%), pericardium (32%), septum (16%), right ventricle (5%), atrium (5%)	NR	Most cases	Most cases	NR	SSA and/or PRRT
Kunz et al. (19)	15/629 (2.38%)	Left ventricle (43%), septum (43%), right ventricle (14%)	8.6 (5.2–17.4)	Most cases	All cases	Lower	NR
Bonsen et al. (20)	6/273 (2.2%)	Septum (33%), left atrium (17%), right ventricle (17%), pericardium (17%), multiple sites (17%)	NR	Most cases	All cases	Lower	SSA (83%)
Calissendorff et al. (21)	4/128 (3.12%)	Left ventricle (25%), right ventricle (25%), multiple sites (50%)	14.2 (7.7–29.8)	All cases	All cases	Lower	SSA (100%)
Carreras et al. (22)	35/4,210 (0.83%)	NR	NR	NR	NR	NR	NR

CNTM, cardiac neuroendocrine tumour metastases; NEN, neuroendocrine neoplasm; NR, not reported; PRRT, peptide receptor radionuclide therapy; SSTR-PET/CT, somatostatin receptor positron emission tomography/computed tomography; SSA, somatostatin analogues; SUV_max_, maximal standardized uptake value.

#### Basic study characteristics

Ten studies including data on 163 patients with CNTM detected by SSTR-PET/CT were selected (Table 1) (13–22). Publication year of the included studies ranged from 2013 to 2024. The studies were performed mainly in European countries (8 out of 10), one in North America and one in Australia. All the studies were retrospective and most of them were single-centre studies (90%).

#### Patients’ characteristics

Patients’ characteristics are summarized in Table 2 (13–22). The number of NEN patients performing SSTR-PET/CT in the included studies ranged from 128 to 4,210. The NEN subtypes performing SSTR-PET/CT were not specified in most of the studies. The number of patients with CNTM detected by SSTR-PET/CT in the included studies ranged from 4 to 35 with a prevalence of male patients in most of the studies and a mean age ranging from 54 to 74 years. When reported, the primary NEN site in patients with CNTM was small bowel in most of the cases and the NEN grading was G1 or G2 in the majority of cases thus representing well-differentiated NEN.

#### Technical aspects

The technical aspects about SSTR-PET/CT are summarized in Table 3 (13–22). The hybrid imaging modality was PET/CT by using low-dose CT or contrast-enhanced CT. When reported the injected PET radiotracer was [^68^Ga]Ga-DOTATATE or [^68^Ga]Ga-DOTATOC with heterogeneous injected activity. The time interval between radiotracer injection and image acquisition ranged from 30 to 75 min. Analysis of PET/CT images was performed using visual/qualitative analysis in all studies considering suspicious for CNTM a focal uptake of the tracer higher than the background. In some studies, semi-quantitative image analysis through the calculation of standardized uptake value (SUV_max_) was carried out. The most frequent radiological imaging methods used for comparison of SSTR-PET/CT findings were CT, cardiac MRI and echocardiography.

#### Main outcome data

Main outcomes from included studies are showed in Table 4 (13–22). The prevalence of patients with CNTM among NEN patients performing SSTR-PET/CT ranged from 0.8 to 3.1% and the most frequent site of CNTM detected by SSTR-PET/CT was the left ventricle even if other cardiac sites and multifocal CNTM were described. The mean SUV_max_ of CNTM ranged from 7.5 to 25.6 and most of the detected CNTM showed high tracer uptake according to the Krenning score (14, 15). An excellent inter-reader agreement in detecting CNTM by using SSTR-PET/CT was reported (19).

Other NEN metastases were present in most of the patients with CNTM, in particular liver, lymph node and bone metastases. However, no significant difference in median overall survival between metastatic NEN with CNTM and metastatic NEN without CNTM was reported (17).

Most of the patients with CNTM were asymptomatic for cardiac symptoms. The performance of other radiological imaging methods in detecting CNTM was lower compared to SSTR-PET/CT. CT showed a very low sensitivity in detecting CNTM (19) and most CNTM were not visualized by echocardiography (15, 17). Kunz et al. (19) reported that CT showed a sensitivity of 19% and a specificity of 100% for the detection of CNTM. Echocardiography was able to detect CNTM only in 7–18% of patients (15, 17). SSTR-PET/CT identified more CNTM than cardiac MRI. In the study of Arnfield et al. (14) 10 patients with CNTM had performed both cardiac MRI and SSTR-PET/CT. At least one CNTM was identified in 9/10 patients who had cardiac MRI and in 10/10 with SSTR-PET/CT; in these 10 patients, 14 CNTM were identified on cardiac MRI and 25 CNTM were identified on SSTR-PET/CT. Notably, when identified on cardiac MRI, CNTM metastases were more accurately localized (14, 15), thus suggesting a complementary role among SSTR-PET/CT and cardiac MRI for the detection and localization of CNTM (Figure 1).

There were very limited data of SSTR-PET/CT compared to other PET/CT methods in detecting CNTM. However, the diagnostic performance of SSTR-PET/CT in this setting is reported as clearly superior compared to [^18^F]FDG PET/CT (17) and similar to that of [^18^F]FDOPA PET/CT (16).

The therapeutic management of CNTM detected by SSTR-PET/CT included therapy with cold somatostatin analogues and/or peptide receptor radionuclide therapy (PRRT) in most of the cases. Few CNTM underwent histological verification of SSTR-PET/CT findings due to the missing necessity for a myocardial biopsy in the overall management of metastatic NEN (13–22). Overall, there were insufficient data about false positive findings for CNTM at SSTR-PET/CT.

### Quantitative analysis (meta-analysis)

**Figure 4:**
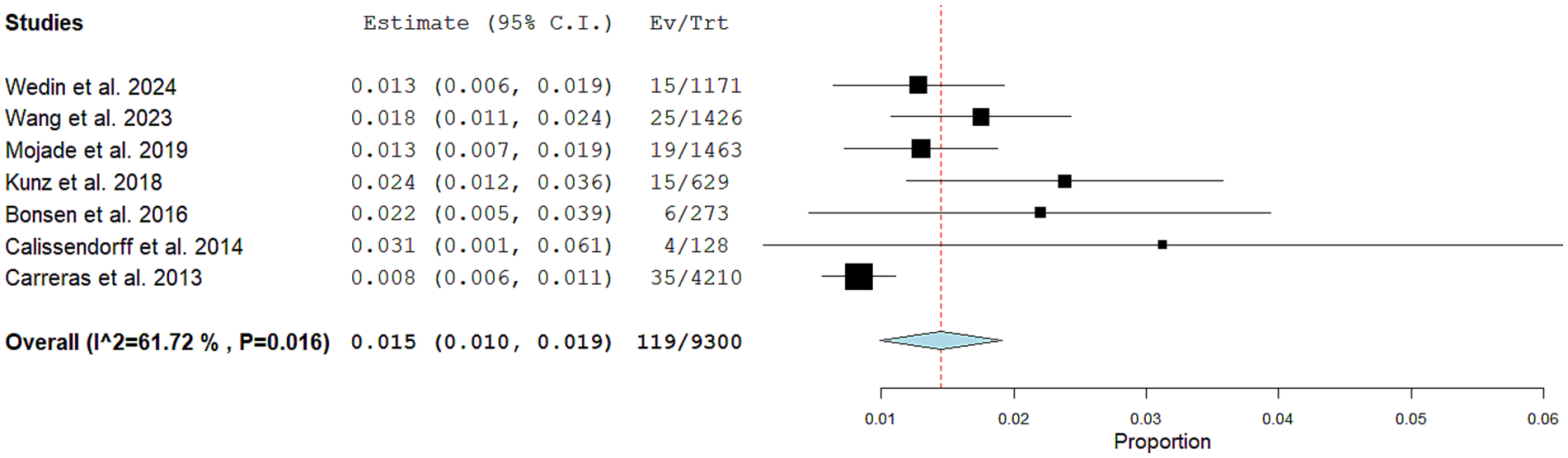
Plots of individual studies and pooled prevalence of patients with CNTM among those with NEN performing SSTR-PET/CT, including 95% confidence intervals (95% CI). The size of the squares indicates the weight of each study. A random-effect model was used for the statistical analysis.

Seven articles including 9,300 NEN patients performing SSTR-PET/CT for staging/restaging and 119 CNTM detected by SSTR-PET/CT were selected for the meta-analysis. The pooled prevalence of patients of CNTM among those performing SSTR-PET/CT for NEN was 1.5% (95% confidence interval: 1.0–1.9%) as illustrated in Figure 4. A moderate statistical heterogeneity was found (*I*^2^: 62%). A significant publication bias was not demonstrated through the Egger’s test (*p* = 0.5).

Performing a subgroup analysis including only the studies published in the last 5 years, a similar pooled prevalence was obtained (1.4%; 95% confidence interval: 1.1–1.8%), respectively, but without statistical heterogeneity (*I*^2^: 0%).

## Discussion

To the best of our knowledge, this is the first systematic review and meta-analysis focused on the detection of CNTM by SSTR-PET/CT. Compared to a previous evidence-based article on CNTM (23), we have focused our review on SSTR-PET/CT only. The reason of this choice is that we hypothesized a better ability of SSTR-PET/CT in detecting CNTM compared to other radiological imaging methods, including CT, cardiac MRI and echocardiography.

Several studies have used SSTR-PET/CT for detecting CNTM, some of them with few cases of CNTM reported (13–22). We have summarized data from the literature through a systematic review and meta-analysis to provide more robust data on the selected topic compared to the single studies included in our analysis (8).

Our analysis shows that the pooled prevalence of patients with CNTM among those performing SSTR-PET/CT is low as expected, but not negligible (1.5%).

We have also demonstrated that the left ventricle is the most common site of CNTM even if localization to other cardiac sites or multifocal CNTM are described (13–22).

Patients with small bowel NEN have the highest tendency to develop CNTM compared to other NEN subtypes (13–22). This finding is quite expected as well-differentiated small bowel NEN are the most frequent NEN.

Most of the patients with CNTM had concurrent metastatic lesions to other organs and this would suggest that CNTM is correlated with the overall metastatic burden (15).

SSTR-PET/CT allows early detection of CNTM due to their SSTR overexpression and most of the patients with these metastatic lesions did not show any cardiac symptoms (13–22). The clinical manifestations of CNTM are nonspecific and depend on their location; moreover, the clinical pattern correlates with the degree of cardiac infiltration (23). However, as patients with CNTM may develop severe clinical consequences with poor clinical outcome, earlier detection of CNTM is relevant to start the most effective treatment and for their management (23).

Advancements in molecular imaging including SSTR-PET/CT, evaluating the SSTR status of NEN, led to a more frequent identification of CNTM compared to other radiological imaging methods (13–22). This finding is not surprising as hybrid imaging, including SSTR-PET/CT in NEN, may detect early functional abnormalities that usually precede morphological changes detected by conventional radiological imaging (3). On SSTR-PET/CT, CNTM lesions typically demonstrate very high target-to-background radiotracer uptake ratio (due to the absent tracer uptake in the heart and the usually high tracer uptake in CNTM), thus facilitating the excellent lesion detection.

Available literature data clearly demonstrate that echocardiography and CT miss most of the CNTM detected by SSTR-PET/CT (15, 17, 19). The main limitations of echocardiography are the operator dependence, the poor tissue contrast and the low performance in detecting small lesions (14). Echocardiography remains the imaging modality of choice to identify and characterize carcinoid heart disease which is a separate clinical manifestation of NEN involving the cardiac valves (15).

About cardiac MRI, this imaging method evaluating mass morphology and tissue characterization, showed excellent accuracy, superior to echocardiography and CT, representing the gold standard imaging method for detection and characterization of cardiac masses (24). Even if cardiac MRI may detect a lower number of CNTM compared to SSTR-PET/CT, it may have a complementary role in this setting providing a more accurate localization and lesion characterization of CNTM compared to SSTR-PET/CT due to its high contrast resolution and tissue characterization; anatomical information is crucial for surgical planning optimization (14). The limitation of SSTR-PET/CT in determining the exact location, size and functional significance of CNTM may be explained by several factors including the relatively limited spatial resolution of PET, the possible misregistration among PET and CT data due to cardiac or respiratory motion and the relatively lower anatomical details provided by low-dose CT compared to MRI (14). Overall, the clinical role of cardiac MRI could be limited to a further delineation of CNTM identified by SSTR-PET/CT rather than its use as frontline screening of CNTM (14, 15).

Even if cardiac MRI remains the preferred radiological imaging method for CNTM characterization, cardiac CT offers a valuable diagnostic alternative, superior to echocardiography, in further evaluation of CNTM detected by SSTR-PET/CT in particular in patients with contraindications to cardiac MRI (16).

Notably, there are no published studies performing SSTR-PET/MRI instead of SSTR-PET/CT in CNTM. Hybrid PET/MRI combining the advantages of PET and MRI could be particularly useful and effective in this setting (3, 25), but its availability in clinical routine is still relatively limited compared to PET/CT (26).

About other PET tracers, compared to [^18^F]FDG PET/CT, SSTR-PET/CT has a higher performance in detecting CNTM (17). This is not surprising as most NEN usually have slow growth and low glucose consumption compared to more aggressive tumours, explaining their usually low [^18^F]FDG uptake (5). Furthermore, unlike SSTR tracers, the heart could be a site of physiological [^18^F]FDG uptake and this is another factor explaining the low sensitivity of [^18^F]FDG PET/CT in detecting CNTM (17).

[^18^F]FDOPA is another PET tracer used NEN imaging, because these tumours can accumulate decarboxylate biogenic amines such as L-DOPA (27). In patients with serotonin-secreting small bowel NEN the ability of [^18^F]FDOPA PET/CT to detect CNTM is expected to be similar compared to SSTR-PET/CT (16, 28, 29).

Clinical management of CNTM includes different therapeutic options. To date, no standard treatment has been defined for CNTM. Specific treatment is often not needed due to the lack of functional cardiac involvement. As most patients with CNTM present concurrent metastatic sites in other organs, a systemic treatment is usually performed. The most frequent therapeutic options used in patients with CNTM in the included studies were cold somatostatin analogues or PRRT, which are safe and justified by the usually high expression of SSTR by neuroendocrine tumours metastases. More invasive treatments were performed only in few cases, mainly due to the functional consequences of CNTM (13–22).

The prognostic implication of CNTM detection by SSTR-PET/CT is still unknown. The clinical significance of CNTM detection could be not high, due to the relatively slow evolution of well-differentiated NEN, leading to a comparable survival in NEN patients without CNTM (17). However, the early CNTM detection would allow the introduction of different treatments in the earliest stages of the disease reducing the functional impact of CNTM with potential better outcome (16).

Some limitations of our analysis should be underlined. The first limitation is the relative low number of included studies, but this number is in line with the infrequent finding of CNTM. The second limitation is the retrospective nature of all the included studies resulting in possible selection bias. Third, we have found a moderate degree of heterogeneity among the included studies, in particular related to patients’ characteristics and technical aspects. Lastly, histopathological verification of SSTR-PET/CT findings in CNTM as gold-standard is reported only in few cases, but invasive diagnosis was not justified in most of the patients included in the selected studies considering their characteristics (most of them were metastatic patients with a known primary NEN and without cardiac symptoms) and the high diagnostic accuracy of SSTR-PET/CT. However, as only few CNTM detected by SSTR-PET/CT were confirmed through histology, false positive cases cannot be excluded.

Based on the data reported in our systematic review and meta-analysis, we would like to suggest the design of a large multicentric and prospective study on the detection of CNTM by SSTR-PET/CT. In particular, further studies are needed to analyze the clinical impact of CNTM detection by SSTR-PET/CT on the outcome of patients with metastatic NEN.

## Conclusion

Evidence-based data demonstrate that SSTR-PET/CT enables early detection of CNTM which are encountered with a pooled prevalence of 1.5% of NEN patients performing SSTR-PET/CT. The ability of SSTR-PET/CT in detecting CNTM is better compared to other radiological imaging methods. Prospective and multicentric studies are warranted to better clarify the impact of CNTM detection by SSTR-PET/CT on overall survival and clinical decision-making in NEN patients.

## Data Availability

The original contributions presented in the study are included in the article/supplementary material, further inquiries can be directed to the corresponding author.

## Footnotes

^1^ http://www.cebm.brown.edu/openmeta/index.html
